# Supplementary material for: Roll-to-roll manufacturing of flexible acetone sensors
Source: PLoS One. 2025 Nov 3;20(11):e0334947. doi: 10.1371/journal.pone.0334947 (PMC12582499; doi:10.1371/journal.pone.0334947)
Supplement: S1 File — (c) IDEs were printed and dried in a semicontinuous R2R process (transport belts for intermittent substrate motion are shown). Fig A2(a) Electrode dimensions. (b) Screen-printed silver electrodes and sensor layout for R2R Printing. Fig A3(a) Electrospraying setup for fabricating MoS2 and SCNT films. The electrosprayed electrodes were around 70 cm long. (b) Homemade gas-sensing system for VOCs measurements. Fig A4 Schematics and photographs of results of various R2R printing layer arrangements. (DOCX) [file pone.0334947.s001.docx]

**Supplementary Information**

**Roll-to-Roll Manufacturing of Flexible Acetone Sensors**

Author Information: Ya-Ching Yu^1^, Nicholas Glassmaker^2^, Ana M. Ulloa^1^, Benson Kunhung Tsai^1^, Amit Barui^1^, Haiyan Wang^1^, and Lia Stanciu^1,3,4*^

Affiliation

^1^School of Materials Engineering, Purdue University, 701 West Stadium Ave., West Lafayette, IN, 47907, USA

^2^ Birck Nanotechnology Center, Purdue University, 1205 W State St, West Lafayette, IN, 47907, USA

^3^School of Biomedical Engineering, Purdue University, 206 S Martin Jischke Dr, West Lafayette, IN, 47907, USA

^4^Bindley Bioscience Center, Purdue University, 1203 W State St, West Lafayette, IN, 47907, USA

* Corresponding author. Department of Materials Engineering, Purdue University, West Lafayette, IN, 47907, USA.

E-mail address: lstanciu@purdue.edu (L. Stanciu).

Phone number: 765-496-3552


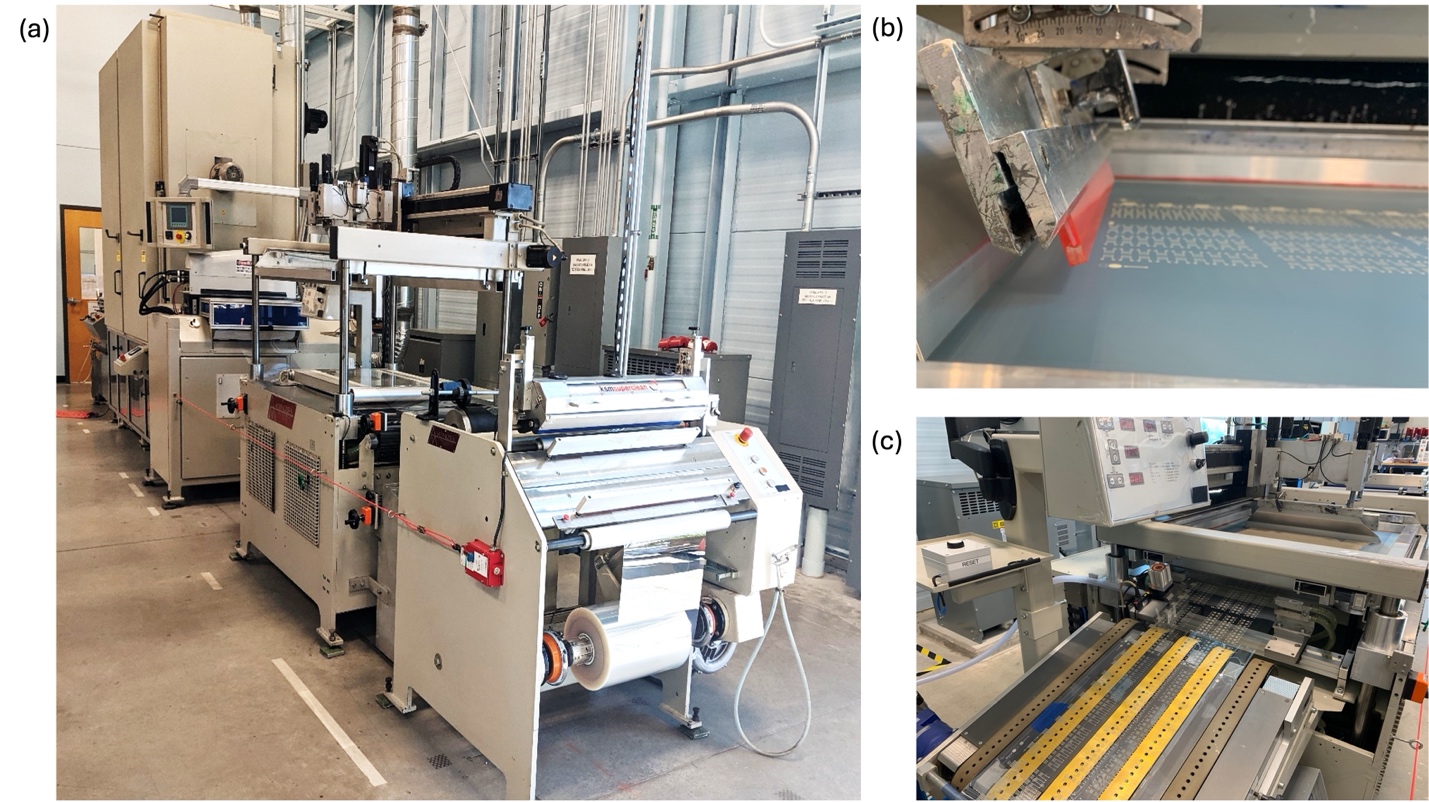


Fig. A. 1 (a) Kinzel roll-to-roll (R2R) screen printer (b) Screen-printing silver paste was applied to the patterned screen shown here to print silver electrodes. (c) IDEs were printed and dried in a semicontinuous R2R process (transport belts for intermittent substrate motion are shown).


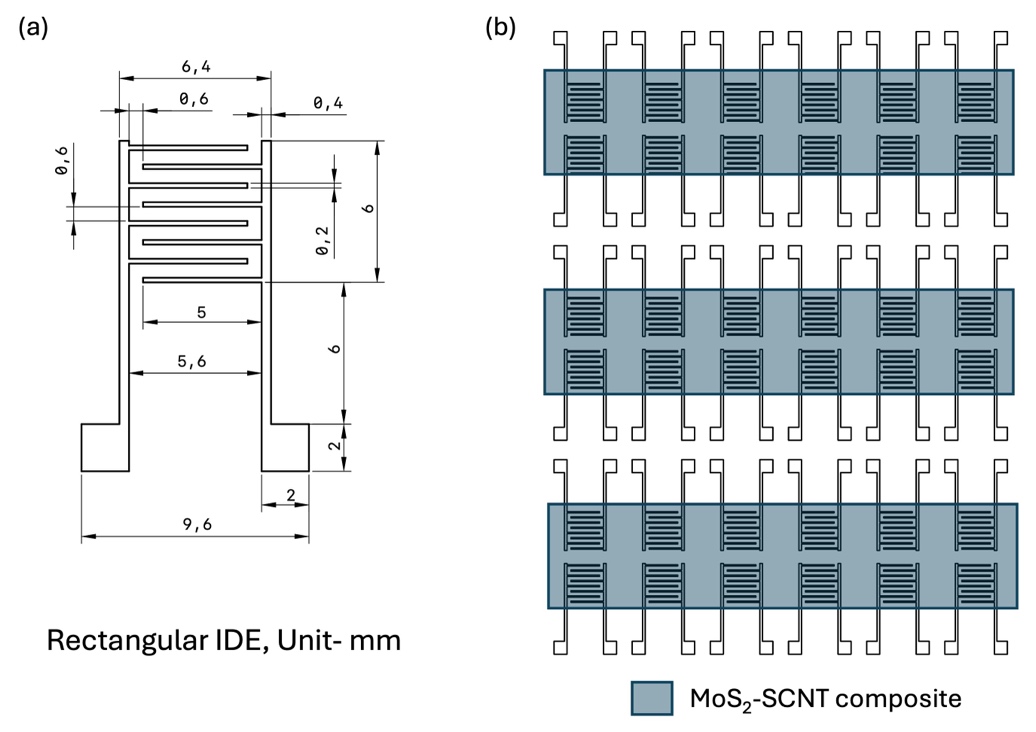


Fig. A. 2(a) Electrode dimensions. (b) Screen-printed silver electrodes and sensor layout for R2R Printing.


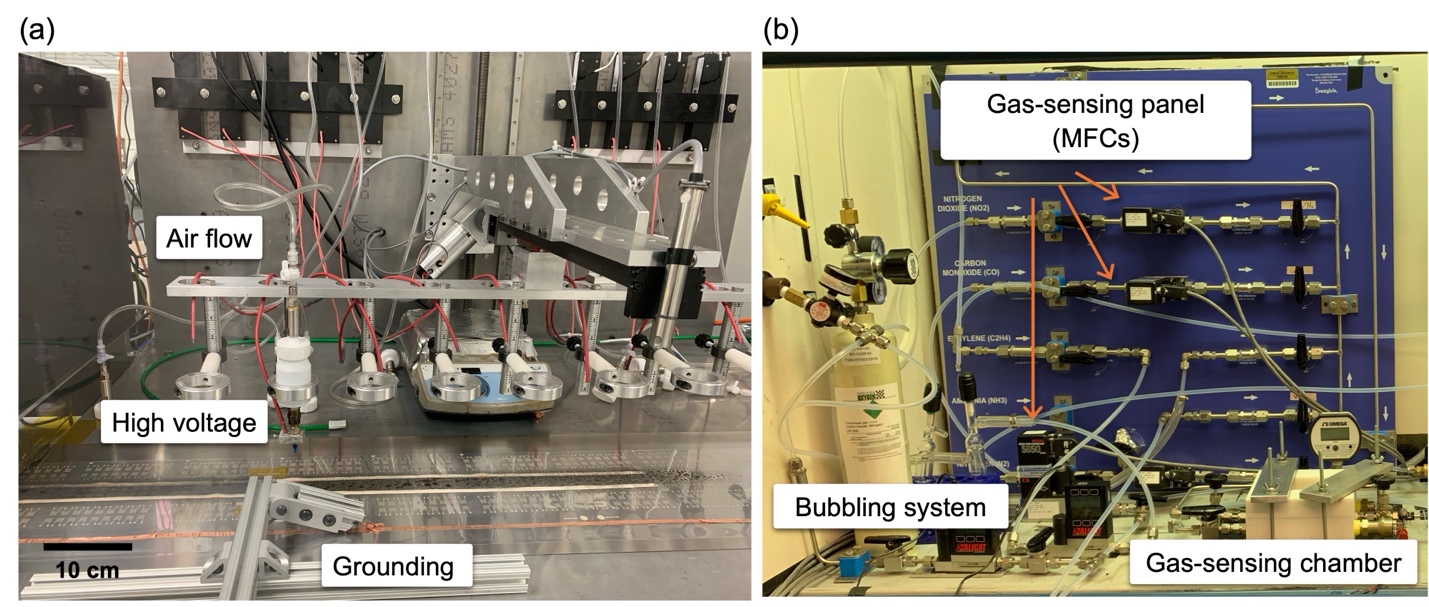


Fig. A.3 (a) Electrospraying setup for fabricating MoS_2_ and SCNT films. The electrosprayed electrodes were around 70 cm long. (b) Homemade gas-sensing system for VOCs measurements.


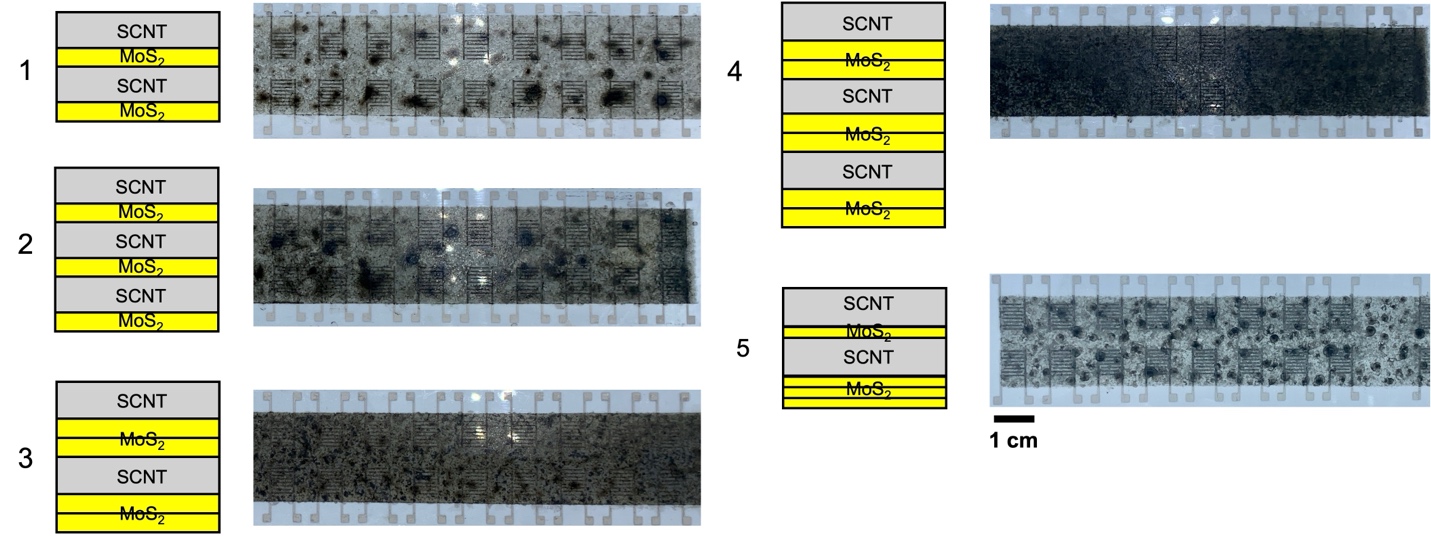


Fig. A. 4 Schematics and photographs of results of various R2R printing layer arrangements.
