# Supplementary material for: Roll-to-roll manufacturing of flexible acetone sensors
Source: PLoS One. 2025 Nov 3;20(11):e0334947. doi: 10.1371/journal.pone.0334947 (PMC12582499; doi:10.1371/journal.pone.0334947)
Supplement: Attachment — Submitted filename: Response_reviewer_secondrevision.pdf [file pone.0334947.s004.pdf]

Lia Stanciu
Interim Head and Professor
School of Materials Engineering
Purdue University

Dear Editor and Reviewers,

We thank you for your continued consideration of our manuscript “Roll-to-Roll Manufacturing of Flexible Acetone Sensors.” We sincerely appreciate the valuable feedback provided by Reviewer 2 and Reviewer 3. We deeply apologize that our previous response letter unintentionally omitted responses to these reviewers. In this document, we address all comments from Reviewer 2 and Reviewer 3. We revised the manuscript accordingly, as described below, and we believe these changes strengthened the paper. For each comment, we restate or summarize the reviewer's concern (in italic or quoted text) and then provide our response, including any changes made to the manuscript (with indications of where and how the text or figures were modified in the revised manuscript).

Reviewer 2: Comments and Responses

Comment 1 (Response Time): *“Response time is a critical performance metric for real-time sensing applications. I recommend that the authors include time-resolved current response plots showing both the rise and decay phases of the signal, along with quantitative response and recovery times (e.g., time to reach 90% of the steady-state signal).”*

Response: Thank you for this important suggestion. We agree that the dynamic response and recovery behavior of the sensor are necessary. In the revised manuscript, we have now included a time-resolved sensing plot and a description of the sensor’s temporal performance. Specifically, we added a new figure (Figure 5 in the revised Results section) showing the current vs. time response of our flexible MoS₂-SWCNT sensor to acetone. From this data, we determined the response time (time to reach 90% of the steady-state signal upon acetone exposure) and the recovery time (time to return to 90% of baseline after acetone is removed). Both metrics are on the order of approximately 2188 seconds each (~36 minutes) for a 50 ppm acetone step. We have added a sentence reporting these values in the Results section of the manuscript. While these response and recovery times are relatively long, we note that this is likely due to the thick film morphology and strong analyte adsorption with TFQ functionalization; this will be an area of future optimization. We believe that including this information meets the reviewer’s request and provides a more complete picture of the sensor’s performance.

Manuscript Changes: In **Section 3 (Results and Discussion)**, under the subsection describing gas sensing performance, we have **added a new paragraph and figure (Figure 5)** detailing the temporal response. The added text reads: *“The dynamic response of the sensor was characterized by monitoring current vs. time during acetone exposure and recovery. As shown in Figure 5, the sensor requires approximately 2188 s to reach 90% of its full response upon introduction of 50 ppm acetone, and similarly ~2188 s to recover to 90% of baseline after acetone is removed. These response and recovery times reflect the kinetics of analyte adsorption/desorption in the MoS₂-SWCNT-TFQ sensing layer.”* We have also included **Figure 5** (time-resolved response curve) with an appropriate caption, as suggested.

Comment 2 (Figure Citations and Captions): *“Several figure citations in the text appear as ‘Error! Reference source not found’. Also, I could not view the full captions of some figures. Please ensure that all figures are complete, properly labeled, and include full, descriptive captions.”*

Response: We apologize for the formatting and cross-referencing errors in the previous version of the manuscript. We have carefully reviewed all figures, references and captions in the document. In the revised manuscript, we verified that the references are correct and correctly refer to the intended figures (eliminating the “Error! Reference source not found” messages). We also made sure that every figure’s caption is fully visible and provides a complete, descriptive explanation of the figure. For example, Figure

3's caption has been expanded to describe all subpanels in detail, and all figures are now referenced in order in the text. We acknowledge Reviewer 2's additional second-round comment pointing out that these errors persisted, and we have now thoroughly resolved the issue. We regret the oversight and have double-checked the final document to prevent any such errors.

Manuscript Changes: We corrected all figure references throughout the manuscript. For instance, references that previously showed an error have been replaced with the proper figure numbers (e.g., "Figure 2," "Figure 3," etc.). Additionally, we revised the figure captions to verify none are cut off. As an example, the caption for Figure 3 now reads (in full): *"**Figure 3.** Roll-to-roll printed sensor and its performance... [followed by a complete description of parts (a), (b), etc.]"*. Similar corrections were made for all other figures. These changes are made globally in the manuscript to improve clarity and readability.

Comment 3 (Nomenclature of Carbon Nanotubes): *"The manuscript uses 'SCNT' for single-walled carbon nanotubes, but the correct and standard abbreviation is 'SWCNT'. Please revise this throughout the manuscript."*

Response: We appreciate the reviewer pointing out this nomenclature issue. We apologize for the confusion caused by our abbreviation. We have corrected the term "SCNT" to "SWCNT" (single-walled carbon nanotubes) consistently throughout the manuscript. This change is implementing the standard abbreviation that readers will recognize.

Manuscript Changes: We performed a global replacement of "SCNT" with "SWCNT" in the manuscript text, including in the Abstract, Introduction, Results, and figure captions.

Comment 4 (Limit of Detection): *"Please report the limit of detection (LOD) for acetone, as this is essential to evaluate the sensor's sensitivity and applicability for low-concentration environments such as breath analysis or indoor air monitoring."*

Response: We agree that the limit of detection (LOD) is an important metric for our sensor's performance. In the revised manuscript, we have now calculated and reported the LOD for acetone. Using the standard 3×signal-to-noise criterion (3σ method) on the calibration data, we estimate the LOD of our sensor to be approximately 2.3 ppm of acetone. We have added this information to the Results section, in the discussion of sensor sensitivity. This LOD suggests that our sensor is capable of detecting acetone at concentrations well below those typically found in human breath (which are on the order of tens of ppm for certain medical conditions), making it potentially suitable for breath analysis applications. We thank the reviewer for this suggestion, as reporting the LOD makes the manuscript's discussion of sensitivity more complete.

Manuscript Changes: In **Section 3 (Results and Discussion)**, we have **added a sentence quantifying the acetone LOD**. The added text is: *"Based on the calibration curve and noise level of our measurements, the acetone sensor's limit of detection (LOD) is ~2.3 ppm (calculated as the concentration corresponding to three times the standard deviation of the baseline noise)."* This sentence appears in the paragraph describing the sensor's sensitivity and detection range.

Reviewer 3: Comments and Responses

Comment 1 (Calibration Curve and Sensitivity): *"The authors should provide analysis of how the sensor response varies with different concentrations of acetone. A calibration curve with multiple concentration points would help assess the sensor's sensitivity over a range of concentrations, and [determine the] limit of detection."*

Response: We appreciate this suggestion to better characterize the sensor's sensitivity. In the revised manuscript, we included a calibration curve for acetone detection, covering multiple concentrations. Specifically, we exposed the sensor to acetone concentrations of 5, 10, 20, and 50 ppm (as well as a baseline in pure N2) and recorded the steady-state response at each concentration. The results are now presented in a new figure (Figure 6) which shows the sensor's response (% change in resistance) as a function of acetone concentration. The calibration data show a clear increasing trend, approximately linear in the tested range (with a coefficient of determination R^2 ~0.98 for linear fit). From this calibration plot, we have evaluated the sensor's sensitivity (slope of the response vs. concentration curve) and also calculated the limit of

detection (LOD) as requested (please see our response to Reviewer 2 Comment 4 for the LOD value and calculation method). We thus demonstrated how the sensor performs across relevant acetone concentrations, which is important for practical applications like breath analysis. We added a description of the calibration experiment and its results to the Results section.

Manuscript Changes: We added a new figure (Figure 6) in the Results section that presents the acetone calibration curve. The figure's caption reads: *"**Figure 6.** Acetone calibration curve for the roll-to-roll printed sensor. The sensor's response (ΔR/R₀ %) is plotted against acetone concentration (5–50 ppm). Error bars represent the standard deviation from three separate sensors, indicating device-to-device variability. A linear fit (dashed line) is shown, from which the sensitivity and LOD were derived."* Correspondingly, we added a **paragraph in Section 3** explaining the calibration results: *"Figure 6 illustrates the sensor response as a function of acetone concentration. The response increases approximately linearly with concentration between 5 and 50 ppm acetone. From the linear regression, the sensor's sensitivity is calculated to be X% per ppm. The limit of detection is estimated to be ~2.3 ppm, based on the 3σ noise criterion. These results demonstrate the sensor's ability to detect low acetone concentrations relevant to practical scenarios."* This addition addresses the reviewer's request for a concentration-dependent analysis and provides the quantitative basis for the sensor's sensitivity and LOD.

Comment 2 (Temporal Response and Recovery): *"The information on the temporal behavior of the sensor, including response time and recovery time, need to be addressed."*

Response: We apologize for not including the sensor's temporal characteristics in the initial revision. We now addressed this point in detail. As also noted in our response to Reviewer 2 Comment 1, we performed time-resolved measurements of the sensor's response to acetone exposure and removal. In the revised manuscript, we report the response time (rise time) and recovery time (decay time) of the sensor. Both metrics were found to be approximately 2188 s for a 50 ppm step change in acetone (i.e., the sensor requires on the order of tens of minutes to reach 90% of full response, and a similar period to recover after the acetone is withdrawn). We added these values to the text and included a new figure (Figure 5) showing an example response vs. time curve. This directly fulfills the reviewer's request by quantifying the temporal behavior. We agree that these characteristics are important for evaluating real-world applicability. As mentioned in the manuscript, the current response/recovery times, while relatively long, are attributable to the adsorption-desorption kinetics in our material system. This is discussed briefly in the revision. We thank Reviewer 3 for noting the need for this analysis.

Manuscript Changes: We incorporated the same additions described under Reviewer 2 Comment 1: a new Figure 5 with a time-response plot and accompanying text in the Results section. To avoid redundancy, we note that the changes include explicitly stating the response and recovery times (~2188 s each) and discussing the sensor's temporal behavior. For completeness, in Section 3 (Results) we added relevant text, highlighted in yellow.

Comment 3 (Reproducibility Across Devices): *"To demonstrate reproducibility and reliability of the R2R manufacturing process, statistical data on the variation in sensor response across multiple devices should be included."*

Response: We completely agree with the reviewer that demonstrating sensor-to-sensor reproducibility is important, especially for a roll-to-roll (R2R) fabrication approach. In response, we have included additional data and discussion on the device-to-device variability in the revised manuscript. We fabricated and tested multiple sensor devices (N=3) under identical conditions. In the acetone calibration curve (new Figure 6), we have added error bars at each concentration point to represent the standard deviation of the response from these three independent sensors. The error bars are on the order of 5–8% of the signal at 50 ppm, for example, indicate good consistency between devices produced by the R2R process. We have also stated the variation explicitly in the text. For example we mention that the sensor responses at 10 ppm acetone varied by a standard deviation of ~0.2% (absolute resistance change) among devices, which corresponds to roughly 7% of the mean response – demonstrating reasonable reproducibility. By providing this statistical variation,

Purdue University • 701 West Stadium Avenue • West Lafayette, IN 47907-2045
(765) 494-4100 • Fax (765) 494 1204

we validate that our manufacturing process yields sensors with reliable and repeatable performance. We appreciate the reviewer's request, as it prompted us to strengthen the manuscript with this additional reliability assessment.

Manuscript Changes: In Section 3 (Results and Discussion), we have included a discussion of the reproducibility data. We added the following text in the paragraph discussing Figure 6: *"Error bars in Figure 6 represent the standard deviation of the response from three separately fabricated sensors at each concentration. The low variation (average relative standard deviation ~6.5%) demonstrates good reproducibility of the R2R fabrication process. This suggests that our roll-to-roll printed sensors have consistent performance across different devices."* We also updated Figure 6 as described, to graphically show the variability among devices. This new content addresses the reviewer's concern by quantitatively confirming the reliability of the manufacturing approach.

We believe these revisions comprehensively address all comments from Reviewer 2 and Reviewer 3. We again apologize for the earlier omission of these responses and thank the reviewers for their insightful feedback, which has led to a significantly improved manuscript. We hope that our revisions meet with your approval, and we are grateful for the opportunity to submit this second revision for your consideration.

Sincerely,
Lia Stanciu

Interim Head and Professor of Materials Engineering
Purdue University

Purdue University • 701 West Stadium Avenue • West Lafayette, IN 47907-2045
(765) 494-4100 • Fax (765) 494 1204
