# Supplementary material for: Roll-to-roll manufacturing of flexible acetone sensors
Source: PLoS One. 2025 Nov 3;20(11):e0334947. doi: 10.1371/journal.pone.0334947 (PMC12582499; doi:10.1371/journal.pone.0334947)
Supplement: Attachment — Submitted filename: Reviewer_response.docx [file pone.0334947.s003.docx]

**Response to Reviewers Questions**

1. What is the mechanism by which TFQ improves the selectivity of acetone sensors?

**Answer.** We thank the reviewer for this important question. The mechanism by which tetrafluorohydroquinone (TFQ) improves selectivity toward acetone involves both chemical interactions and electronic effects. TFQ molecules are known to interact non-covalently with carbon-based nanomaterials such as single-walled carbon nanotubes (SCNTs) via π–π stacking and hydrophilic interactions. When TFQ is deposited on the MoS₂–SCNT composite film, it forms a uniform thin layer that modulates the surface chemistry of the sensor.

Acetone has a relatively high dipole moment (2.69 D) compared to other tested VOCs such as ethanol or hexane, which strengthens its interaction with the TFQ layer (see Fig. 3(h) in the manuscript). This strong interaction leads to a more pronounced charge transfer between acetone and the TFQ-functionalized sensing surface. Specifically, acetone, acting as an electron-withdrawing molecule, facilitates the transfer of electrons from TFQ to the p-type SCNTs. This results in a reduction in hole concentration and an increase in the sensor's resistance, which increases the selectivity and signal strength for acetone.

We added text in the revised manuscript to describe this mechanism.

2. For the experiment section in page 6, the authors mention that MoS_2_-SCNT films were then functionalized by casting 1, 2, 5, 10, 20, 30, and 50 μL drops of TFQ. What is the area of casting? Is it a single IDE or multiple IDEs prepared by R2R large-area preparation?

**Answer.** We thank the reviewer for the comment. In response, we have added more information on the TFQ Functionalization in Section 2, Experimental Section on Page 6 as follows:

TFQ solution was dropped cast on top of the MoS_2_-SCNT film, which was electrosprayed on IDEs. The MoS_2_-SCNT film on each single IDE was then functionalized by casting 1, 2, 5, 10, 20, 30, and 50 μL drops of TFQ and dried in the hood as an ultrathin film. The as-prepared sensors could be stored in a desiccator for the following measurement.

3. In Fig. 2(d), authors use three IDEs to test the current values under the VOC and N2. Is the final value the average of the current values of the three electrodes?

**Answer.** We thank the reviewer for the comment. In response, we have added more information on the TFQ Functionalization in Section 3, Results and discussion on Page 8 as follows:

To evaluate their performance for conductometric VOC detection, interdigitated electrodes (IDEs) coated with MoS_2_-SCNT networks were tested in initial experiments. Three IDEs were placed inside a flow cell at room temperature, and exposed to a constant gas flow rate of 0.5 L/min. Electrodes were connected to a potentiostat to monitor changes in current, initially being exposed to nitrogen for 10 minutes, followed by a 5-minute exposure to 20 vt% of various VOC analytes mixing with 80 vt% of nitrogen. The observed change in current reflects a temporary alteration in the sensor’s electronic states caused by charge transfer interactions between the MoS_2_-SCNT composite and adsorbed VOC molecules. For instance, acetone, an electron-withdrawing molecule, captures electrons from the conduction band of the n-type MoS_2_ upon adsorption, reducing carrier density and leading to a decrease in current [1]. When the MoS_2_-SCNT film is exposed to VOC analytes, the molecules are adsorbed onto its nanoporous network structure and withdraw electrons from the film, further decreasing the current. The results demonstrated that the nanoporous structure effectively captures VOC analytes, although the sensor's specificity was found to be limited, as shown in Fig. 2 (e).

4. According to the definition of "Response" mentioned by the authors on page 7, the response is only positive when the current value of the electrode in the VOC analyte is higher than the current value in N2. In Fig. 2 (e), the response of all VOC analytes is positive, indicating that the current value of the electrode in the VOC analyte is higher than that in N2. This is inconsistent with the author's description of ' the MoS2-SCNT-coated IDEs showed a rapid decrease in relative current '.

**Answer.** We thank the reviewer for the comment. The response is based on the current change, so we added the absolute value to the response formula, as follows:

$$Response \left( \% \right)= \frac{\Delta I}{I_{0}} \times100= \frac{\left| {I_{g}- I}_{0} \right|}{I_{0}} \times100$$

In response, we have added more information in Section 3, Results and Discussion on as follows:

The observed change in current reflects a temporary alteration in the sensor’s electronic states caused by charge transfer interactions between the MoS_2_-SCNT composite and adsorbed VOC molecules. For instance, acetone, an electron-withdrawing molecule, captures electrons from the conduction band of the n-type MoS_2_ upon adsorption, reducing carrier density and leading to a decrease in current [1]. When the MoS_2_-SCNT film is exposed to VOC analytes, the molecules are adsorbed onto its nanoporous network structure and withdraw electrons from the film, further decreasing the current. The results demonstrated that the nanoporous structure effectively captures VOC analytes, although the sensor's specificity was found to be limited, as shown in Fig. 2 (e).

5. As a paper reporting on acetone sensors, the author should provide the detection sensitivity of the sensor. For example, the current response of the sensor at different acetone concentrations, such as 10% -90% concentration.

**Answer.** We thank the reviewer for this suggestion. Our current sensor platform was optimized and tested using a standard concentration of 20 vt% acetone in nitrogen to evaluate relative responses and material performance. As noted in the manuscript (Section 3, Results and Discussion), the primary goal was to demonstrate uniformity and scalability of the roll-to-roll (R2R) manufacturing process and validate TFQ functionalization as a route to increase selectivity to acetone, as lack of selectivity is a common problem of many VOC sensors.

However, we agree that a sensitivity study across a broader concentration range is valuable and plan to include this in our next study. At present, our sensor system’s configuration and detection circuitry are optimized for high-concentration exposures (≥20 vt%). We are currently developing a new study that is focusing on low-concentration detection and calibration, including signal amplification and testing against concentrations down to single-digit percentages.

We have added this clarification and a note on future sensitivity characterization work in the revised manuscript (Conclusion section).

**Reference**

1. J. Cao *et al.*, “Recent development of gas sensing platforms based on 2D atomic crystals,” *Research*, 2021.
